# Height, adiposity and body fat distribution and breast density in young women

**DOI:** 10.1186/bcr3228

**Published:** 2012-07-13

**Authors:** Joanne F Dorgan, Catherine Klifa, John A Shepherd, Brian L Egleston, Peter O Kwiterovich, John H Himes, Kelley Pettee Gabriel, Linda Van Horn, Linda G Snetselaar, Victor J Stevens, Bruce A Barton, Alan M Robson, Norman L Lasser, Snehal Deshmukh, Nola M Hylton

**Affiliations:** 1 Fox Chase Cancer Center, 333 Cottman Avenue, Philadelphia, PA 19111, USA; 2 University of California, San Francisco, 1600 Divisadero Street, San Francisco, CA 94115, USA; 3 Johns Hopkins Hospital, 600 N. Wolfe Street, Baltimore, MD 21287, USA; 4 University of Minnesota, 1300 S. Second Street, Minneapolis, MN 55454, USA; 5 University of Texas Health Science Center, 1616 Guadalupe Street, Austin, TX 78701, USA; 6 Northwestern University Feinberg School of Medicine, 680 N. Lake Shore Drive, Chicago, IL 60611, USA; 7 University of Iowa, 200 Hawkins Drive, Iowa City, IA 52242, USA; 8 Kaiser Permanente Center for Health Research, 3800 N. Interstate Avenue, Portland OR 97227, USA; 9 University of Massachusetts Medical School, 333 South Street, Shrewsbury, MA 01545, USA; 10 Children's Hospital, 200 Henry Clay Avenue, New Orleans, LA 70118, USA; 11 New Jersey Medical School, 65 Bergen Street, Newark, NJ 07107, USA

## Abstract

**Introduction:**

Breast density is one of the strongest risk factors for breast cancer, but determinants of breast density in young women remain largely unknown.

**Methods:**

Associations of height, adiposity and body fat distribution with percentage dense breast volume (%DBV) and absolute dense breast volume (ADBV) were evaluated in a cross-sectional study of 174 healthy women, 25 to 29 years old. Adiposity and body fat distribution were measured by anthropometry and dual-energy X-ray absorptiometry (DXA), while %DBV and ADBV were measured by magnetic resonance imaging. Associations were evaluated using linear mixed-effects models. All tests of statistical significance are two-sided.

**Results:**

Height was significantly positively associated with %DBV but not ADBV; for each standard deviation (SD) increase in height, %DBV increased by 18.7% in adjusted models. In contrast, all measures of adiposity and body fat distribution were significantly inversely associated with %DBV; a SD increase in body mass index (BMI), percentage fat mass, waist circumference and the android:gynoid fat mass ratio (A:G ratio) was each associated significantly with a 44.4 to 47.0% decrease in %DBV after adjustment for childhood BMI and other covariates. Although associations were weaker than for %DBV, all measures of adiposity and body fat distribution also were significantly inversely associated with ADBV before adjustment for childhood BMI. After adjustment for childhood BMI, however, only the DXA measures of percentage fat mass and A:G ratio remained significant; a SD increase in each was associated with a 13.8 to 19.6% decrease in ADBV. In mutually adjusted analysis, the percentage fat mass and the A:G ratio remained significantly inversely associated with %DBV, but only the A:G ratio was significantly associated with ADBV; a SD increase in the A:G ratio was associated with an 18.5% decrease in ADBV.

**Conclusion:**

Total adiposity and body fat distribution are independently inversely associated with %DBV, whereas in mutually adjusted analysis only body fat distribution (A:G ratio) remained significantly inversely associated with ADBV in young women. Research is needed to identify biological mechanisms underlying these associations.

## Introduction

The breast is comprised of adipose tissue and dense fibroglandular tissue, and women with a high percentage dense breast area (%DBA) or absolute dense breast area (ADBA) measured by mammography are at an increased risk of breast cancer. In a meta-analysis, risk was increased more than fourfold for women with the highest %DBA [1]. The percentage dense breast volume (%DBV) and absolute dense breast volume (ADBV) measured by magnetic resonance imaging (MRI) and other three-dimensional modalities are similarly positively associated with breast cancer risk [2].

The association of body composition with breast density has been studied extensively. Adiposity is strongly inversely associated with %DBA [3-13] and %DBV [12-16] in both premenopausal women and postmenopausal women. Adiposity generally is also inversely associated with ADBA in postmenopausal women [6-8,10,13], but the association is less consistent in premenopausal women, with inverse [3,7,10] and direct [8,9,11] associations reported. The association of adiposity with ADBV is more often reported to be direct [12,14-16], although inverse [13] and null [17] associations also have been reported. Height is positively associated with %DBA in some studies [3,7,18], but it is not associated with ADBA in most studies [3,6,10,11,18]. Only one previous study has evaluated the association of height with %DBV and ADBV [16], and in that study height was positively associated with both measures of breast density.

Most earlier studies estimated breast density as %DBA and ADBA from mammographic images and included older premenopausal and postmenopausal women. Because of the sensitivity of the young breast to radiation, few studies have evaluated the association of adiposity and height with breast density in girls and young women. Nonetheless, breast density measured by mammographic parenchymal pattern was inversely associated with body mass index (BMI) and percentage truncal fat in a study of 25 to 35 year olds [19]. Novotny and colleagues used dual-energy X-ray absorptiometry (DXA) to measure %DBV and ADBV in 10-year-old to 16-year old girls, and found that several DXA measures of adiposity were significantly inversely associated with %DBV but positively associated with ADBV [20]. Percentage fat mass was the strongest predictor of %DBV and explained 67% of its variability. Using MRI to measure %DBV and ADBV in young women 15 to 30 years old, Boyd and colleagues also found that adiposity assessed by body weight was significantly inversely associated with %DBV but positively associated with ADBV [16]. Height, on the contrary, was significantly positively associated with both %DBV and ADBV.

Height is positively associated with breast cancer risk particularly after menopause [21,22]. The association of adiposity with breast cancer is complex and differs over the life-course. Whereas obesity is positively associated with breast cancer risk after menopause, it is inversely associated with risk before menopause [21,22]. Furthermore, obesity at a young age confers long-term protection against breast cancer that extends past menopause [23]. However, few studies have explored associations of height and adiposity with %DBV and ADBV in young women. We therefore used data from the Dietary Intervention Study in Children Follow-up Study (DISC06) to evaluate associations of height, adiposity and body fat distribution with %DBV and ADBV in a sample of women 25 to 29 years old.

## Materials and methods

### Design

The Dietary Intervention Study in Children (DISC) was a multicenter randomized controlled clinical trial sponsored by the National Heart, Lung, and Blood Institute to test the safety and efficacy of a dietary intervention to reduce serum low-density lipoprotein cholesterol (LDL-C) in children with elevated LDL-C. The trial's design and results have been described previously [24-29]. Briefly, between 1988 and 1990, 663 healthy, pre-pubertal, 8-year-old to 10-year-old children with elevated LDL-C, including 301 girls, were recruited into the DISC at six clinical centers and were randomized to a behavioral dietary intervention or usual care control group. The planned intervention continued until 1997 when the mean age of participants was 16.7 years. From 2006 to 2008, when participants were 25 to 29 years old, the DISC06 follow-up study was conducted to evaluate the longer-term effects of the diet intervention on biomarkers associated with breast cancer risk in DISC female participants. Assent was obtained from DISC participants and informed consent was obtained from their parents/guardians prior to randomization. Informed consent was obtained from participants again prior to the DISC06 follow-up visit. The original and follow-up DISC protocols were approved by Institutional Review Boards at all participating centers.

### Participants

All female DISC participants were invited to participate in the DISC06 and 260 (86.4%) of the 301 females originally randomized took part. Women who were pregnant or breastfeeding at or within 12 weeks before the visit (*n *= 30) and those who had breast augmentation or reduction surgery (*n *= 16) were not eligible for inclusion in the current analysis, leaving a total of 214 women. Analyses were restricted to women with complete MRI, DXA and anthropometric data to allow direct comparisons of associations of different measures of adiposity with breast density. Consequently, otherwise eligible women were excluded if they had technically unacceptable/missing MRI images (*n *= 26), technically unacceptable/missing DXA images (*n *= 6), technically unacceptable/missing MRI and DXA images (*n *= 6), or missing waist circumference (*n *= 1). Finally, one participant who was highly influential in several models and had extreme values for %DBV and ADBV (first percentile), for BMI (98th percentile) and for waist circumference (99th percentile) was excluded, leaving a total of 174 participants for inclusion in analyses.

### Data collection

For the follow-up study, each female participant attended a single visit at one of the six DISC clinics between 2006 and 2008. Visits were scheduled to take place in the luteal phase of the menstrual cycle whenever possible, and 85% of visits took place within 14 days of onset of next menses. All data for a participant were collected on the same day except 24-hour dietary recalls, which were collected over 2 weeks following the visit. Additionally, if a participant had not fasted, blood collection was re-scheduled for the following day whenever possible. Data were collected by staff masked to treatment assignment. A centralized data collection training session was held before initiation of data collection to train and certify individuals responsible for data collection.

Participants completed several questionnaires on demographic characteristics, on medical, reproductive and menstrual histories, on medication use, and on health habits including smoking.

### Anthropometry

Height was measured using a stadiometer, weight was measured on an electronic or beam balance scale, and waist circumference was measured at the level of the uppermost lateral border of the right iliac crest using an anthropometric measuring tape. Each measurement was made twice on each participant. A third measurement was taken if the first two measurements were not within allowable tolerances (0.5 cm for height and waist circumference and 0.2 kg for weight) and the two closest values were averaged.

### Body composition

**Figure 1:**
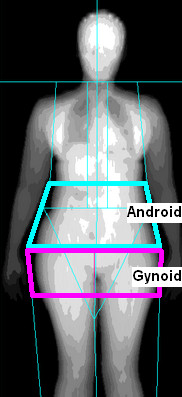
**Dual-energy X-ray absorptiometry whole-body scan**. Android region outlined in blue and gynoid region outlined in pink.

Body composition was measured using clinical DXA protocols. Scans were acquired of the lumbar spine (L1 to L4), proximal femur, and whole body at default scan speeds on Hologic (Hologic, Inc. Bedford, MA, USA) and GE Lunar (General Electric/Lunar, Madison, WI, USA) systems. All DXA image data were processed centrally at the University of California, San Francisco (UCSF) by trained staff coordinated by one of the investigators (JAS). Centralized analyses were performed using the manufacturers' software (Hologic version 12.4; Lunar Prodigy version 11.4) on each scan following the guidelines of the International Society for Clinical Densitometry [30]. Whole-body scans also report subregional values, including the arms, legs, trunk, head, android and gynoid regions (Figure 1). Because of the substantial correlation between the android and gynoid fat masses, relative fat distribution was characterized as the android:gynoid (A:G) fat mass ratio in the analyses. Total adiposity was characterized by the percentage whole-body fat mass estimated by the ratio of whole-body fat mass to whole-body total mass.

Different DXA systems were used at the six DISC clinical centers. Systems of the same make and model were cross-calibrated to one reference site using a set of static calibration objects (that is, phantoms). Inter-manufacturer calibration for spine and hip was accomplished using the *in vivo *universal standardization equations [31,32], whereas whole-body results were cross-calibrated using equations derived by UCSF from unpublished data. Furthermore, device-specific spine and whole-body phantoms were scanned routinely throughout the course of the study to allow correction for any calibration drifts. To insure accuracy and uniformity of data acquisition at the different clinical sites, all DXA personnel were trained by UCSF personnel on the protocol, patient positioning, data transfer, and phantom scanning procedures. The clinical center was not certified to recruit study participants until test data on five volunteers met UCSF's quality assurance standards. Over the course of this study, 10 scans out of the total 666 scans acquired were excluded by UCSF because of artifacts, motion, or poor positioning.

### Breast density

Breast density was measured using noncontrast MRI. Equipment standards were consistent with American College of Radiology guidelines for breast MRI [33] and required that imaging be performed using a whole-body MRI scanner of 1.5 Tesla or higher field strength and a dedicated breast imaging radiofrequency coil. A standard image-acquisition protocol was prescribed consisting of two pulse sequences performed in both the transaxial and coronal orientations with a 32 to 40 cm field of view for bilateral coverage: a three-dimensional fast gradient echo sequence without fat suppression, and a three-dimensional fast gradient echo sequence with fat suppression.

To insure accuracy and uniformity of data acquisition at the different clinical centers, MRI technologists at the sites were individually trained (by CK) to recognize and correct failures due to incomplete fat suppression, motion artifacts, and inadequate breast coverage. In addition, acceptable image quality on three volunteers was required for site certification. Participant scans that were inaccurate due to artifacts, motion or technique were excluded (*n *= 21).

All MRI image data were processed at UCSF by the same investigator (CK) using customized software to identify the chest wall-breast tissue boundary and skin surface, and to separate breast fibroglandular and fatty tissue using a segmentation method based on fuzzy C-means clustering [34]. Fuzzy C-means segmentation was performed using fat-suppressed images; nonfat-suppressed images were used when incorrect or failed segmentation occurred due to poor fat suppression. In problematic cases that could not be segmented with automated fuzzy C-means methods, manual delineation was used. Total volumes of fibroglandular and fatty tissue were computed separately for each breast. %DBV was measured as the ratio of fibroglandular volume to total volume of the breast. ADBV also was used.

### Statistical analysis

%DBV and ADBV were transformed to natural logarithms to improve normality. Adiposity measures were transformed to *z*-scores relative to the observed distribution so that a unit change in each measure represents a change in one standard deviation (SD) unit. To evaluate associations with adiposity, linear mixed-effects models were fit by maximum likelihood with robust standard errors separately for %DBV and ADBV. The clinic was included in all models as a random effect; all other variables were included as fixed effects. Models were fit using backward stepwise elimination to ensure that we had adequate power to detect associations of interest. To facilitate direct comparison of associations of anthropometric and DXA-derived measures of adiposity with %DBV and ADBV, the same covariates were included in all adjusted models. Variables that were significantly associated (*P *< 0.05) with either %DBV or ADBV in analysis of anthropometric or DXA measures of body composition were included in all adjusted models. These models included terms for race (white, nonwhite), education (attended college), smoking status (current, former/never), duration of hormonal contraceptive use and parity. Regressions of %DBV and ADBV on height also were adjusted for current weight.

To allow comparison with the extant literature, which generally does not adjust for childhood BMI when evaluating associations of adult adiposity with breast density, results are presented with and without adjustment for childhood BMI. The BMI at 8 to 10 years of age, expressed as a *z*-score relative to the Centers for Disease Control 2000 Growth Charts [35] to account for changes in BMI with age in childhood and adolescence, was added to adjusted models. Treatment group, age at visit, family history of breast cancer, age at menarche, menstrual cycle day modeled as a cubic spline, past-year leisure physical activity, and alcohol ingestion also were considered potential confounders, but were not retained in final multivariable adjusted models.

The presence of interaction was evaluated by testing the significance of the cross-product term of variables in a model that also included their main effects. Mulitcollinearity was evaluated by calculating variance inflation factors from simple linear regression models [36]. Potentially highly influential observations were initially identified using leverage-versus-squared residual plots [37]. Models were compared including and excluding these observations, and one observation that changed results sufficiently to change interpretation of the data was excluded from the final analysis. Percentage differences in %DBV and ADBV associated with a SD difference in anthropometric and DXA body composition measures were estimated from models as:

Δ%=(exp(β)-1)×100

All tests of statistical significance were two-sided. All analyses were conducted using STATA version 12.0 (College Station, TX, USA) and SAS version 9.2 (Cary, NC, USA).

## Results

Participant characteristics are shown in Table 1. Their mean ± SD age was 27.2 ± 1.0 years (range 24.9 to 29.7 years) and the majority was white. The women were well educated, with 67.2% having a bachelors or graduate degree. Most women (73.0%) were nulliparous. At the time of the visit, 58.6% of the women were using hormonal contraceptives and 35.1% had used them formerly. Among current and former hormone users, the mean duration of use was 5.6 ± 3.5 years. Almost one-quarter of participants smoked cigarettes at the time of the visit, with an average of 8.5 ± 7.6 cigarettes per day. Participants' mean BMI was 25.0 kg/m^2^; 25.9% were overweight (25 ≤ BMI < 30) and another 16.1% were obese (BMI ≥ 30). The participants' mean total fat mass measured by DXA was 25.2 kg, which was 35.4% of the total mass (lean plus fat). Their mean A:G ratio was 0.38.

**Table 1:** Participant characteristics

Descriptive characteristic	*n*	Mean (standard deviation) or %
Age (years)	174	27.16 (1.02)
Duration hormone use by current and former users (years)	163	5.64 (3.52)
Number of cigarettes per day by current smokers	40	8.53 (7.55)
Body mass index *z*-score at 8 to 10 years old	174	0.20 (0.89)
Race		
White	156	89.66
Nonwhite	18	10.34
Education		
High school, vocational or technical school	18	10.34
Some college	39	22.41
Bachelor degree	92	52.87
Graduate degree	25	14.37
Number of full-term pregnancies		
0	127	72.99
1	28	16.09
2 to 4	19	10.92
Hormone use		
Never	11	6.32
Former	61	35.06
Current	102	58.62
Smoking status		
Never	97	55.75
Former	37	21.26
Current	40	22.99
Anthropometric measures		
Height (cm)	174	165.09 (6.35)
Waist (cm)	174	82.67 (12.36)
Body mass index (kg/m^2^)	174	24.97 (4.82)
DXA body composition measures		
Total fat (%)	174	35.40(8.80)
Android:gynoid fat mass ratio	174	0.38 (0.12)
Breast density measures		
Percentage dense breast volume (%)	174	28.15 (20.39)
Absolute dense breast volume (cm^3^)	174	104.67 (71.28)

Thirty-nine women who were not recently pregnant or breastfeeding and did not have breast reduction or augmentation surgery, and consequently were eligible to be included in analyses, were excluded because they were missing or had technically unacceptable breast density or anthropometric or DXA body composition measures. These women had a larger mean waist circumference compared with women included in analysis (92.0 ± 15.7 cm vs. 82.7 ± 12.4 cm; *P *= 0.001), but otherwise they did not differ significantly on the characteristics included in Table 1.

Anthropometric and DXA measures of body composition and body fat distribution were correlated (Table 2). BMI and percentage fat mass were highly correlated (Spearman *r *= 0.84; *P *< 0.001). BMI and percentage fat mass also were correlated with body fat distribution measured by waist circumference (Spearman *r *= 0.71 to 0.73; *P *< 0.001) and A:G ratio (Spearman *r *= 0.69 to 0.72; *P *< 0.001). All measures of adult adiposity and body fat distribution were also positively correlated with the BMI *z*-score at 8 to 10 years of age (Spearman *r *= 0.34 to 0.56; *P *< 0.001), although less strongly than with concurrently measured BMI.

**Table 2:** Spearman correlations of anthropometric and dual-energy X-ray absorptiometry body composition measures

	Height (cm)	BMI (kg/m^2^)	Waist (cm)	Total fat (%)	A:G ratio	BMI *z*-score at 8 to 10 years old
Height (cm)	1.00					
BMI (kg/m^2^)	-0.19	1.00				
Waist (cm)	< 0.01	**0.71**	1.00			
Total fat (%)	-0.15	**0.84**	**0.73**	1.00		
A:G ratio	-0.16	**0.69**	**0.66**	**0.72**	1.00	
BMI *z*-score at 8 to 10 years old	0.05	**0.56**	**0.37**	**0.42**	**0.34**	1.00

A:G, android:gynoid fat ratio; BMI, body mass index. Bold correlations are statistically significant at *P *< 0.001.

Height was significantly positively associated with %DBV in the adjusted analysis (Table 3). For each SD increase in height, %DBV increased by 18.7% (*P *= 0.002) in the fully adjusted model. Height, however, was not significantly associated with ADBV in unadjusted or adjusted analysis (Table 4).

**Table 3:** Percentage dense breast volume differences associated with difference in anthropometric and body composition measures

	Unadjusted^a^				Adjusted^b^				Also adjusted for childhood or young adult BMI^c^			
	
	% Diff	95% CI	*P *value	*R* ^2d^	% Diff	95% CI	*P *value	*R* ^2^	% Diff	95% CI	*P *value	*R* ^2^
Adult anthropometric measures												
Height (cm)	3.7	-13.8, 24.8	0.70	< 0.01	21.0	9.4, 33.8	< 0.001	0.55	18.7	6.7, 32.1	0.002	0.57
BMI (kg/m^2^)	-50.2	-55.0, -44.8	< 0.001	0.49	-50.2	-55.4, -44.4	< 0.001	0.54	-45.1	-51.5, -37.8	< 0.001	0.56
Waist (cm)	-51.9	-58.8, -43.8	< 0.001	0.38	-51.5	-59.7, -41.6	< 0.001	0.47	-44.4	-51.0, -37.0	< 0.001	0.56
Adult DXA measures												
Total fat (%)	-50.8	-55.0, -46.4	< 0.001	0.53	-50.1	-55.0, -44.7	< 0.001	0.55	-44.4	-49.6, -38.7	< 0.001	0.60
A:G ratio	-52.3	-55.6, -48.8	< 0.001	0.57	-52.0	-54.5, -49.5	< 0.001	0.60	-47.0	-48.5, -45.4	< 0.001	0.67
Childhood BMI												
BMI *z*-score at 8 to 10 years old	-40.0	-47.0, -32.0	< 0.001	0.22	-41.7	-47.5, -35.2	< 0.001	0.34	-17.3	-22.4, -11.9	< 0.001	0.56

Percentage differences (%Diff) in percentage dense breast volume associated with a one-standard-deviation difference in anthropometric and dual-energy X-ray absorptiometry (DXA) body composition measures. Results are also interpretable as the excess relative ratio. To convert to the relative ratio (RRatio), use the formula: RRatio = (%Diff + 100)/100. A:G ratio, android:gynoid fat ratio; BMI, body mass index; CI, confidence interval. ^a^Estimates from six linear mixed-effects models including clinic as a random effect and anthropometric and body composition variables as fixed effects. Anthropometric and body composition variables are modeled separately without mutual adjustment. ^b^Estimates from six linear mixed-effects models as described for unadjusted plus including race, education (attended college), smoking status, duration of hormone use, and parity as fixed effects. Model for height also includes weight as a fixed effect. ^c^Estimates from six linear mixed-effects models as described for adjusted plus including BMI *z*-score at 8 to 10 years old as a fixed effect in adult anthropometric and DXA models and young adult BMI in the childhood BMI model. ^d^Proportion of variance explained by model.

**Table 4:** Absolute dense breast volume differences associated with difference in anthropometric and body composition measures

	Unadjusted^a^				Adjusted^b^				Also adjusted for childhood or young adult BMI^c^			
	
	% Diff	95% CI	*P *value	*R* ^2d^	% Diff	95% CI	*P *value	*R* ^2^	% Diff	95% CI	*P *value	*R* ^2^
Adult anthropometric measures												
Height (cm)	-8.9	-22.2, 6.8	0.25	0.01	-2.5	-12.6, 8.7	0.65	0.19	-5.6	-15.5, 5.4	0.31	0.26
BMI (kg/m^2^)	-20.4	-29.6, -10.0	< 0.001	0.07	-20.8	-29.8, -10.7	< 0.001	0.17	-6.8	-18.9, 7.0	0.32	0.25
Waist (cm)	-23.7	-37.7, -6. 7	0.009	0.08	-24.7	-39.5, -6.3	0.011	0.20	-15.6	-32.1, 4.8	0.12	0.28
Adult DXA measures												
Total fat (%)	-24.6	-33.9, -14.1	< 0.001	0.12	-23.7	-32.9, -13.1	< 0.001	0.20	-13.8	-24.1, -2.3	0.02	0.28
A:G ratio	-26.9	-35.4, -17.3	< 0.001	0.14	-26.6	-34.2, -18.2	< 0.001	0.23	-19.6	-27.1, -11.2	< 0.001	0.30
Childhood BMI												
BMI *z*-score at 8 to 10 years old	-28.4	-37.3, -18.3	< 0.001	0.13	-30.0	-35.9, -23.5	< 0.001	0.25	-27.0	-32.9, -20.7	< 0.001	0.25

Percentage difference (%Diff) in absolute dense breast volume associated with a one-standard-deviation difference in anthropometric and dual-energy X-ray absorptiometry (DXA) body composition measures. Results are also interpretable as the excess relative ratio. To convert to the relative ratio (RRatio), use the formula: RRatio = (%Diff + 100)/100. A:G, android:gynoid fat ratio; BMI, body mass index; CI, confidence interval. ^a^Estimates from six linear mixed-effects models including clinic as a random effect and anthropometric and body composition variables as fixed effects. Anthropometric and body composition variables are modeled separately without mutual adjustment. ^b^Estimates from six linear mixed-effects models as described for unadjusted plus including race, education (attended college), smoking status, duration of hormone use, and parity as fixed effects. Model for height also includes weight as a fixed effect. ^c^Estimates from six linear mixed-effects models as described above for adjusted plus including BMI *z*-score at 8 to 10 years old as a fixed effect in adult anthropometric and DXA models and young adult BMI in the childhood BMI model. ^d^Proportion of variance explained by model.

All anthropometric and DXA measures of adiposity and body fat distribution were significantly inversely associated with %DBV in unadjusted and adjusted analyses (Table 3). In adjusted models that did not include childhood BMI, each SD increase in adult BMI, percentage fat mass, waist circumference and A:G ratio was associated significantly with an approximately 50.1 to 52.0% decrease in %DBV (all *P *< 0.001). The BMI *z*-score at 8 to 10 years of age was independently and significantly inversely associated with %DBV; after adjusting for childhood BMI in addition to other covariates, a unit increase in each measure of adult adiposity and body fat distribution was significantly associated with a 44.4 to 47.0% decrease in %DBV (all *P *< 0.001).

All anthropometric and DXA measures of adiposity were also significantly inversely associated with ADBV in unadjusted and adjusted analyses that did not include childhood BMI (Table 4). In these adjusted analyses, each SD increase in adult BMI, percentage fat mass, waist circumference and A:G ratio was associated significantly with a 20.8 to 26.6% decrease in ADBV (all *P *≤ 0.011). However, childhood BMI was independently and significantly inversely associated with ADBV, and adjustment for the BMI *z*-score at 8 to 10 years of age attenuated associations of adult adiposity and body fat distribution with ADBV. After adjustment for childhood BMI and other covariates, only the DXA measures of percentage fat mass and A:G ratio remained significant; a SD increase in each was associated significantly with a 13.8% and 19.6% decrease in ADBV, respectively (all *P *< 0.05).

As noted above, measures of adiposity and body fat distribution were correlated. However, even though mutual adjustment attenuated associations, the percentage fat mass and A:G ratio each remained significantly (*P *< 0.001) inversely associated with %DBV (Table 5). Similarly, BMI and waist circumference were independently inversely associated with %DBV. In contrast, although A:G ratio remained significantly inversely associated with ADBV after adjustment for percentage fat mass, the percentage fat mass was no longer significantly associated after adjustment for the A:G ratio. Adjusted for percentage fat mass, a SD increase in the A:G ratio was associated significantly with an 18.5% decrease in ADBV (*P *< 0.001). Even though measures of total adiposity and body fat distribution were highly correlated, the highest variance inflation factor for individual variables included in models was 2.98 and the highest model average variance inflation factor was 1.60, indicating that multicollinearity was not a serious problem [36].

**Table 5:** Percentage and absolute dense breast volume differences with difference in mutually adjusted anthropometric and body composition measures

	Percentage dense breast volume^a^				Absolute dense breast volume^a^			
	
	% Diff	95% CI	*P *value	*R* ^2b^	% Diff	95% CI	*P *value	*R* ^2^
Anthropometric measures								
BMI (kg/m^2^)	-29.1	-48.2, -3.1	0.03	0.61	13.4	-19.1, 58.8	0.47	0.29
Waist circumference (cm)	-28.6	-49.4, -0.7	0.06		-22.5	-47.9, 15.2	0.21	
DXA measures								
Total fat (%)	-26.1	-34.1, -17.3	< 0.001	0.71	-2.4	-16.7, 14.3	0.76	0.30
A:G ratio	-37.2	-40.3, -34.0	< 0.001		-18.5	-27.8, -7.9	0.001	

Percentage difference in percentage and absolute dense breast volume with a one-standard-deviation difference in mutually adjusted anthropometric and dual-energy X-ray absorptiometry (DXA) measures. Results also are interpretable as the excess relative ratio. To convert to the relative ratio (RRatio), use the formula: RRatio = (%Diff + 100)/100. A:G, android:gynoid fat ratio; BMI, body mass index; CI, confidence interval. ^a^Estimates from two linear mixed-effects models including clinic as a random effect and anthropometric and body composition variables, race, education (attended college), smoking status, duration of hormone use, parity, and BMI *z*-score at 8 to 10 years old as fixed effects. ^b^Proportion of variance explained by model.

Women in our study participated in a controlled clinical trial of a diet intervention during childhood and adolescence. Approximately one-half were randomly assigned to the intervention group and one-half to the usual care control group. Tests for interaction did not indicate that group assignment modified associations of height or anthropometric and DXA measures of adiposity with %DBV or ADBV (data not shown).

## Discussion

Adiposity and body fat distribution were strongly inversely associated with %DBV in this study of 25-year-old to 29-year-old women. Associations were weaker for ADBV but remained significant in adjusted analysis for the DXA measures of percentage fat mass and A:G ratio. The percentage fat mass and A:G ratio were independently inversely associated with %DBV, but in mutually adjusted analysis only the A:G ratio remained significantly inversely associated with ADBV. Height was positively associated with %DBV but was not associated with ADBV. Research is needed to identify the physiological mechanisms underlying these associations.

Our study had several strengths. Data collection was performed using standardized procedures by trained personnel and numerous quality controls were in place to ensure data integrity. %DBV and ADBV were measured by MRI, which is a tomographic rather than projection technique, and therefore is not impaired by high parenchymal breast density, making it especially effective for younger women with dense breast tissue. MRI can easily distinguish dense fibroglandular breast tissue from fatty breast tissue with a high degree of contrast and gives three-dimensional information not provided by mammography. Even though %DBA and %DBV are highly correlated [16,34,38], volumetric measures of percentage breast density have been reported to be more strongly associated with breast cancer risk compared with area measures [2]. Body composition was measured by DXA, which yields accurate and precise estimates of adiposity [39,40], and estimation of the A:G ratio enabled discrimination of associations of total adiposity and body fat distribution with %DBV and ADBV. Data were available from the original DISC trial on childhood BMI *z*-score, which was an independent predictor of breast density in our analysis.

Our study also had some limitations. All participants had elevated LDL-C as children when they were randomized in the DISC and met several additional eligibility criteria, which could reduce generalizability of the findings. In an analysis by Boyd and colleagues, LDL-C was significantly inversely associated with %DBA after adjusting for age and BMI [41], but in an analysis by Tamburrini and colleagues, the association was no longer significant after adjusting for waist circumference [42]. We observed a nonsignificant inverse correlation of LDL-C with %DBV in the current analysis (*r *= -0.12, *P *= 0.11) after adjustment for age and BMI. LDL-C was also not correlated with ADBV in analysis adjusted for age and BMI. Moreover, only 14 participants (8.0%) included in analyses had high LDL-C levels at follow-up visits based on National Cholesterol Education Program guidelines [43], and none were using cholesterol-lowering medications. We measured body composition by DXA, which does not distinguish metabolically distinct visceral abdominal fat from subcutaneous fat. DXA measures of android fat, however, are highly correlated with estimates of visceral fat obtained by MRI [39]. Because total fat mass, android fat mass and gynoid fat mass were highly correlated, it was not possible to evaluate their independent associations with %DBV and ADBV. The A:G ratio measures body fat distribution and was used instead.

Thirty-nine otherwise eligible women were excluded from the current analysis because they had missing or technically unacceptable MRI or whole-body DXA images or waist circumference. These women had a larger mean waist circumference compared with women included in the analysis but did not differ on other characteristics evaluated. Technically unacceptable MRIs were a consecutive series from a single clinic where participants' mean waist circumference was higher compared with the other clinics. Women with technically unacceptable whole-body DXA scans also had larger waist circumferences compared with other women, which could have been due to difficulties fitting into the DXA scanning region. Missing MRI and DXA scans were not related to waist circumference.

BMI is an indirect measure of body fat, and, although it is highly correlated with direct measures of percentage fat mass and used extensively as a measure of adiposity, has known limitations. The relationship of BMI to percentage fat mass is nonlinear and differs between men and women [44]. Furthermore, BMI tends to overestimate body fat in lean individuals with high muscle mass. Even so, in our analysis associations of BMI and percentage fat mass with %DBV were comparable. Associations of ADBV with BMI were slightly weaker than percentage fat mass, but interpretations generally were similar except when adjusted for childhood BMI *z*-score. Childhood BMI *z*-score was more strongly correlated with adult BMI than with adult percentage fat mass, and adjustment for childhood BMI *z*-score attenuated the association of ADBV with adult BMI to a greater extent than percentage fat mass, such that only the association of percentage fat mass with ADBV remained statistically significant.

Total adiposity, estimated by BMI or percentage fat mass, was significantly inversely associated with %DBV in our study. These results are consistent with earlier studies in premenopausal women [3-5,7,9-11,14,16], postmenopausal women [4-8,10,13,14] and combined premenopausal and postmenopausal women [12,15] that report inverse associations with adiposity regardless of whether percentage density was measured by area [3-13] or volume [12-16]. In contrast, the association of adiposity with absolute density varies across studies, possibly due to differences in populations studied and measurement of ADBA versus ADBV. Although ADBA and ADBV are positively correlated (*r *~ 0.33), these correlations are considerably weaker compared with %DBA and %DBV (*r *= 0.76) [38]. The majority of studies in postmenopausal women report inverse associations of ADBA with BMI and percentage fat mass that are weaker compared with %DBA [6-8,10,13]. The association of ADBA with adiposity in premenopausal women is less consistent, with significant inverse [3,7,10] and direct [8,9,11] associations reported. In contrast, the association of adiposity with ADBV is more often reported to be direct [12,14-16], although inverse [13] and null [17] associations also have been reported. None of these studies adjusted for childhood BMI, which as described above attenuated inverse associations of adult BMI and percentage fat mass with ADBV in our study.

Mutual adjustment for percentage fat mass and A:G ratio unveiled important differences in associations of total adiposity and body fat distribution with %DBV and ADBV. Specifically, whereas percentage fat mass and body fat distribution were independently and inversely associated with %DBV, only body fat distribution was associated with ADBV in mutually adjusted analysis. Women with more central or android fat mass relative to gynoid fat mass had significantly less ADBV. Our findings for the A:G ratio are consistent with earlier reports of inverse associations for abdominal fat measured by waist circumference, waist-to-hip ratio, DXA percentage trunk fat or computed tomography abdominal fat area with %DBV and %DBA [4,8,10,11,13] and with ADBV and ADBA [4,8,10,13] in premenopausal and postmenopausal women. Similar to our findings, Tseng and Byrne reported independent inverse associations of total and abdominal adiposity with %DBA [11], whereas only abdominal adiposity remained significantly associated with %DBA in models that included measures of total and abdominal adiposity in the study by Woolcott and colleagues [13]. In the latter study, mutual adjustment attenuated associations of both total adiposity and abdominal adiposity such that neither remained significantly associated with ADBA.

Inverse associations of the A:G ratio with %DBV and ADBV that we observed could reflect inverse associations of android fat mass or direct associations of gynoid fat mass with these measures of breast density. Accumulation of abdominal visceral fat is associated with an adverse metabolic profile that includes insulin resistance [40,45] and suppression of the growth hormone axis [45,46]. Most studies do not support an association of insulin resistance with %DBA or ADBA that is independent of adiposity [47,48], but growth hormone is the primary secretagogue for insulin-like growth factor-1, which is positively associated with %DBA and ADBA in premenopausal women [49-51]. Lower levels of insulin-like growth factor-1 in association with abdominal obesity could thus potentially underlie inverse associations of the A:G ratio with %DBV and ADBV. Alternatively, visceral fat varies inversely with estrogens [52], and lower estrogens in association with a high A:G ratio also could potentially explain its inverse association with %DBV and ADBV.

Height was significantly positively associated with %DBV in adjusted analysis, but it was not associated with ADBV in adjusted or unadjusted analysis. Boyd and colleagues previously reported significant positive associations of height with %DBA [3], %DBV [16] and ADBV [16] in premenopausal women, and suggested that growth hormone, which also was positively associated with %DBV [16], might mediate this association. Height is positively associated with %DBA in some studies [3,7,18] but not in others [6,10,11]. In contrast, height generally is not associated with ADBA [3,6,10,11,18], although a significant but weak positive correlation was reported in a study of young women [16]. Height was also positively associated with ADBV in that study, which is in contrast to the null association we observed.

Body composition was evaluated in association with breast density in young women in two prior studies. Similar to us, in a study of 25 to 35 year olds, Furberg and colleagues reported an inverse association of BMI and percentage truncal fat with percentage breast density assessed by mammographic parenchymal patterns [19]. Findings from our study and the study in young women by Boyd and colleagues [16] on associations of height and adiposity with %DBV were also similar, but differed for ADBV. The reason for these differences is unclear. However, our studies differed in important ways. Age is an important determinant of %DBV and ADBV [16], and participants in our study were 25 to 29 years old compared with 15 to 30 years old in the study by Boyd and colleagues [16]. Some of the younger girls in the latter study may not have attained final adult height or completed breast development at the time of assessments. Girls had to be at or above the fifth percentile for height and within the fifth to 95th percentile of weight for height when 8 to 10 years old to be eligible for DISC, and consequently our study. The mean height of Boyd and colleagues' participants was similar to ours at follow-up visits (165.8 ± 5.9 cm vs. 165.1 ± 6.3 cm), but our participants tended to weigh more (60.6 ± 10.5 kg vs. 68.0 ± 13.4 kg) [16]. Boyd and colleagues used body weight as a measure of adiposity, whereas we used BMI and percentage fat mass measured by DXA. However, our results were unchanged by substituting weight for BMI and adjusting for height as Boyd and colleagues did (data not shown). Breast MRIs were performed mostly in the luteal phase of the menstrual cycle in our study but in the follicular phase in Boyd and colleagues' study [16]. Although variation in %DBV and ABDV across the menstrual cycle generally is less than 10% [53,54], larger fluctuations have been reported [53,55] and could have contributed to differences in findings. Boyd and colleagues' [16] estimates of %DBV and ADBV were substantially larger than ours, and technical differences in estimating %DBV and ADBV also could have contributed to differences in observed associations. Finally, models in the two studies differed in terms of the covariates included.

The association of adiposity with breast cancer is complex. Whereas obesity is inversely associated with breast cancer in premenopausal women, it is positively associated with risk in postmenopausal women [21,22]. The inverse association we observed between adiposity and %DBV and ADBV is consistent with obesity's protective effect for breast cancer in young women. Childhood obesity confers long-term protection against breast cancer risk [23] and has been reported to be inversely associated with breast density measured by mammographic parenchymal patterns [56] and %DBA [57]. In our analysis, the BMI *z*-score at 8 to 10 years of age was inversely associated with %DBV and ADBV, and adjustment for childhood BMI attenuated associations of adult BMI with ADBV by 67%, but with %DBV by only 10%. The nondense compartment of the breast is composed of adipose tissue, and not surprisingly %DBV - which is the ratio of dense-to-dense plus nondense breast volume - was more strongly associated with current adiposity than childhood adiposity. However, childhood BMI was a strong independent predictor of ADBV and explained much of the association of adult BMI with ADBV. The mechanisms underlying these associations are currently unknown. However, childhood adiposity and associated metabolic, hormonal and inflammatory factors potentially could program breast development at a critical time, leading to life-long changes in breast morphology, breast density and breast cancer risk. Differences in unmeasured childhood adiposity could also contribute to inconsistencies in the literature on the associations of adult adiposity with ADBA and ADBV noted above.

## Conclusion

In young women, adiposity and body fat distribution are significantly and independently inversely associated with %DBV. Associations with ADBV are weaker and more complex. Total adiposity measured as percentage fat mass and body fat distribution are significantly inversely associated with ADBV, but with mutual adjustment only body fat distribution remained significant. Height is significantly positively associated with %DBV. Additional research is needed to identify the biological mechanisms underlying these associations.

## Abbreviations

ADBA: absolute dense breast area; ADBV: absolute dense breast volume; A:G ratio: android:gynoid fat mass ratio; BMI: body mass index; %DBA: percentage dense breast area; %DBV: percentage dense breast volume; DISC: Dietary Intervention Study in Children; DISC06: Dietary Intervention Study in Children Follow-up Study; DXA: dual-energy X-ray absorptiometry; LDL-C: low-density lipoprotein cholesterol; MRI: magnetic resonance imaging; UCSF: University of California, San Francisco.

## Competing interests

The authors declare that they have no competing interests.

## Authors' contributions

JFD conceived of the study, participated in its design and data analysis, and drafted the manuscript. CK and NMH quantified %DBV and ADBV from MRI images and participated in drafting the manuscript. JAS quantified body composition from DXA images and participated in drafting the manuscript. BLE and JHH participated in data analysis and in drafting the manuscript. SD participated in data analysis. POK, LVH, and VJS participated in study design, data collection and in drafting the manuscript. LGS, AMR, NLL, and BAA participated in study design and data collection. KPG participated in data collection and in drafting the manuscript. All authors read and approved the final manuscript.
